# Endocranial volume increases across captive generations in the endangered Mexican wolf

**DOI:** 10.1038/s41598-022-12371-6

**Published:** 2022-05-17

**Authors:** Leila Siciliano-Martina, Margot Michaud, Brian P. Tanis, Emily L. Scicluna, A. Michelle Lawing

**Affiliations:** 1 Department of Biology, Texas State University, 154 Supple Science Building, San Marcos, TX 78666 USA; 2 Interdisciplinary Ecology and Evolutionary Biology Program, Texas A&M University, College Station, TX USA; 3 Department of African Zoology, Royal Museum for Central Africa, Tervuren, Belgium; 4 Department of Integrative Biology, Oregon State University-Cascades, Bend, OR USA; 5 Department of Ecology, Environment and Evolution, School of Life Sciences, La Trobe University, Melbourne, Australia; 6 Department of Ecology and Conservation Biology, Texas A&M University, College Station, TX USA

**Keywords:** Ecology, Conservation biology

## Abstract

Endangered animals in captivity may display reduced brain sizes due to captive conditions and limited genetic diversity. Captive diets, for example, may differ in nutrition and texture, altering cranial musculature and alleviating constraints on cranial shape development. Changes in brain size are associated with biological fitness, which may limit reintroduction success. Little is known about how changes in brain size progress in highly managed carnivoran populations and whether such traits are retained among reintroduced populations. Here, we measured the endocranial volume of preserved Mexican wolf skulls across captive generations and between captive, wild, and reintroduced populations and assessed endocranial volume dependence on inbreeding and cranial musculature. Endocranial volume increased across captive generations. However, we did not detect a difference among captive, wild, and reintroduced groups, perhaps due to the variability across captive generations. We did not find a relationship between endocranial volume and either inbreeding or cranial musculature, although the captive population displayed an increase in the cross-sectional area of the masseter muscle. We hypothesize that the increase in endocranial volume observed across captive generations may be related to the high-quality nutrition provided in captivity.

## Introduction

The relative endocranial volume of the vertebrate skull varies within and between populations of a species^1–3^. Given its intricate relationship with brain size and structure, endocranial volume has been linked to differences in behavior, performance, and fitness^2,4^. Changes in endocranial volume may have significant consequences for the viability of a population^1,5,6^. Within captive populations, endocranial volumes have been documented to increase [e.g.,^7^], decrease [e.g.,^7–9^], and in some cases show no discernable difference [e.g.,^10,11^]. Most frequently, the reported changes occurring in captivity have been related to a reduction in endocranial volume [e.g.,^7–9^], which has been attributed to improper diets, socially and environmentally depauperate enclosures, or lack of stimulating enrichment activities^5,12,13^. The nature of this relationship has been poorly documented in carnivoran species, particularly those that are intensively managed and bred for reintroduction initiatives.

Measurements of endocranial volume provide a useful and widely used proxy for brain size^14,15^. These measurements are often correlated with social behaviors and other traits related to learning, memory, problem-solving, and behavioral flexibility^1,4,16^ and may also influence traits directly related to fitness, including longevity and fecundity^6,8^. Changes in endocranial volume are not necessarily proportional across all brain structures^16,17^. For example, domestic dogs show disproportionate decreases to the neocortex and olfactory bulb associated with a smaller endocranial volume (roughly 30%) compared to their wild counterparts [^18^; although see^19^]. Disproportionate changes in endocranial volume may significantly influence cognition and important sensory functions^16,20^.

Within wild vertebrate populations, environmental variables have been linked to intraspecific variation in brain size, including seasonality, environmental severity, habitat complexity, and urbanization^3,15,21^. However, brain tissue is metabolically expensive to maintain; therefore, intraspecific increases in brain size are primarily thought to occur under intense selection pressure or when ample resources are available, which can enable increased development without risking other aspects of fitness^3,5,8^. Resource limitations are thought to constrain brain size in wild populations^3,8^. However, in managed populations, where ample nutrient-rich diets may be readily and consistently available, and where energetically expensive activities are eliminated (e.g. foraging costs or predator avoidance), enhanced endocranial development may be achievable. However, the relative impact of changes to endocranial volume occurring in captivity has yet to be explored in populations that have been reintroduced to wild conditions.

Previous studies have demonstrated a change in brain volume occurring in captivity, including a decrease in brain volume among captive fish^8,12,22^, waterfowl^23^, and a variety of mammalian taxa including rodents^16,24,25^, artiodactyls^26,27^, perissodactyls^18,28,29^ and carnivorans^7,30^, as well as an increase in brain volume associated with captive Asiatic lions (although the opposite trend was documented among sub-Saharan lions in this study)^7^. Such changes have been attributed to small population sizes (e.g., inbreeding depression and/or genetic drift), pleiotropy associated with breeding for docility, and relaxed selection, where maintaining a large relative brain size may be quickly lost in the absence of selection pressures in natural environments^8,17,31^. While brain size is generally heritable^6,15^, plastic changes related to captive husbandry may arise within a single captive generation^8,12,14^.

The shape of bone structures is constrained, at least partially, by the musculature of a given anatomical region^32,33^. For example, braincase morphology is determined in part by cranial musculature involved in food intake and vocalization^33,34^. This influence of muscle tissue on underlying bone structures may have a considerable impact on the phenotypic evolution of dietary specialists. In particular, large hypercarnivorous mammals (whose diet is composed of at least 70% vertebrate prey)^35,36^ evolved enhanced cranial musculature, which enables them to restrain and kill their prey and process mechanically tough dietary items^33,34^ alongside distinct skull morphologies^37–39^. However, captive carnivores frequently consume a comparatively soft diet compared to what is available in the wild^40,41^, which may lead to changes in skull shape and functionality^42–44^ and may potentially alleviate constraints on internal cranial structures as a byproduct. Although the discussion of a captive diet often focuses on nutritional quality, nutritional quality may not be sufficient to ensure morphological change does not occur in captive individuals^40,42,45^. Likewise, the development of brain tissue is facilitated by high-nutrient diets, where malnourished animals may experience poor brain development and decreased endocranial volume^46,47^. The differential nutritional quality available in captivity may therefore lead to differences in brain development.

The diets of captive Mexican wolves (*Canis lupus baileyi*), for example, may vary between facilities; however, husbandry manuals for the species suggest that 90–95% of their captive diet should be composed of a high-grade meat-based dry domestic dog food (or a similar dry food formulated for exotic canids) and the remaining 5–10% of their diet may include supplemental meat for enrichment^48,49^; whereas, up to 93% of their wild diet is composed of elk (*Cervus elaphus*) and other large ungulates^50,51^. Although dry dog food diets are likely to accommodate the nutritional needs of the animals, the composition of such food is quite different from their wild prey, due in part to the large percentage of cereals other grains often found in dry dog foods^52,53^. This may lead to changes in cranial musculature and bite force, resulting in observable shifts in cranial morphology^43,54^. For example, when comparing captive and wild canid populations, hypercarnivorous species, including Mexican wolves, display a greater change in cranial morphology than less carnivorous species, potentially suggesting a shift in the cranial musculature of captive hypercarnivorous canids^55^.

Here, we investigated the relative endocranial volume of Mexican wolves across captive generations and among captive, wild, and reintroduced populations. Given the small founding size of their captive population (only 7 individuals) and the population bottleneck the species experienced prior to the formation of the captive population^56,57^ as well as the unique conditions provided in captivity (e.g., enclosures, nutrition, enrichment)^8,15,46,47^, we expected to see a cumulative change in endocranial volume across captive generations. In particular, we expected to find a strong relationship between inbreeding coefficients associated with the captive population and a change in brain size. Likewise, given the difference in diet texture provided in captivity, we expected to find a change in cranial musculature between captive, wild, and reintroduced wolves and across captive generations. By quantifying the endocranial volume of a managed population and comparing it to wild and reintroduced populations, this study explores the long-term viability of captive-breeding and helps illuminate the intraspecific variation that may be available to populations that are released from resource scarcity.

## Methods

Mexican wolves (*Canis lupus baileyi*) are a highly managed, endangered population^57^. These animals are endemic to Mexico and the southwestern United States, although they were hunted to near extinction by 1976^56,58^. Mexican wolves were successfully bred within zoos and wildlife sanctuaries from a founding population of just 7 individuals^56,57^. By 1998, individuals from this captive-bred population were reintroduced to portions of its native range in Arizona and New Mexico^56,57^.

We assessed the endocranial volume of 53 captive, 22 wild, and 12 reintroduced Mexican wolves (Appendix A). We distinguished among these groups using the Mexican wolf studbook^57^ and museum records. Individuals from the historic wild population were collected between 1900 and 1950, prior to any reintroduction attempts (*c.* 1998). Captive individuals included only animals that spent the majority (at least 60%) of their first year in the captive environment (either a zoo or enclosed wildlife sanctuary). Reintroduced individuals included animals that had a captive-lineage but spent the majority of their first year (at least 60%) in the wild (as assessed from the studbook). Given that the brain and the cranium do not fully develop until seven months of age in canids^59^, we used exclusively adult specimens for all analyses. Maturity was established via the absence of cranial sutures and deciduous teeth and verified by the captive studbook when possible. To avoid improper estimates of endocranial volume, we only used specimens that were completely intact and had no discernable cranial damage.

To measure endocranial volume, we poured glass beads, 2 mm in diameter, into the foramen magnum of preserved specimens until the beads reached the occipital condyles of the skull, at which point, we gently tapped the skull several times to ensure that the cranial cavity was completely filled. This method is frequently used, easily accessible, and delivers comparable estimates of endocranial volume as measures derived from a cranial CT scan^14^. We used a funnel to remove the beads and recorded their weight in grams to the nearest one hundredth using an electronic balance. To ensure the closest possible measurement, this process was repeated three times for each skull. Based on the mean measure of the three estimates, we calculated a measurement error of less than 1%. We converted the mean mass estimate into volume by developing a calibration curve. Beads were measured into precise volumes of 0, 25, 75, 100, 150, and 200 mL and weighed (to the nearest one hundredth of a gram) resulting in a regression line (y = 0.6692x + 0.7927) used to estimate the volume of each endocranial skull measure.

We assessed the dataset for allometry associated with endocranial volume by regressing it against skull length, a proxy for body size in canids^35,60^ that is frequently used in analyses of brain-to-body size in mammals^7,14^. Each skull was photographed in the ventral view by the same individual to maintain consistent specimen orientation. The length of each skull, from the prosthion to the inion, was measured (in one hundredths of a millimeter) using TPSdig232 software^61^. Using a simple linear regression, we detected allometry (r^2^ = 0.15, F = 15.62, p = 1.6e^−4^; *P*-value significance: 0.01–0.05*, 0.001–0.01**, 0–0.001***). In an effort to minimize the influence of allometry, we extracted the regression residual for each specimen in the dataset and used these values in the remaining analyses.

Given that captive Mexican wolves are known to display a significant difference in cranial length and width when compared to wild populations^62^, we assessed allometrically corrected residuals of endocranial volume (hereafter relative endocranial volume) relative to the skull centroid size, which accounts for both skull length and width. To calculate the centroid size, we photographed the ventral view of the cranium for each specimen and applied 36 homologous landmarks that capture the entire length and width of the skull (Figure S1) using TPSdig232^61^. Based on this landmark scheme, the centroid size of each cranium was calculated as the square root of the sum of squared differences between each point and the geometric center (centroid) of the landmarks^63^. To test whether there are differences in relative endocranial volume among captive, wild, and reintroduced populations, we performed ANOVAs and Tukey’s Honest Significant Difference (HSD) to evaluate pairwise comparisons.

All reintroduced individuals and most captive individuals in the dataset were associated with a recorded studbook number, which allowed us to extract data regarding the specimen’s age, housing history, and lineage (Appendix A). To assess whether relative endocranial volume had any age-related effects, we extracted the age of each captive specimen (in days) and performed a simple linear regression of age to relative endocranial volume. There was no clear statistical relationship between ages and relative endocranial volume within our sample (r^2^ = − 0.01, F = 0.15, p > 0.05), therefore, all specimens (seven months and older) were pooled for the remaining analyses. Using a Welch two sample t-test of male and female specimens, we did not detect any statistically clear sexual dimorphism associated with the relative endocranial volume (t = 1.48, p > 0.05), similar to other canid species [^19^; although see^59^]; therefore, the sexes were pooled in all remaining analyses. Using the *pedigree* package^64^, we calculated the inbreeding coefficients (F) associated with captive individuals from the data provided in the studbook. We used the inbreeding coefficients as independent variables in simple linear regressions to explore the relative impact of inbreeding upon endocranial volume among captive individuals.

For each captive specimen, we also estimated the number of captive generations (generations removed from the wild) in their family tree. We calculated captive generations by adding one to the mean value of the generations experienced by both the sire and the dam^65^. In total, our dataset included individuals from 1.5 to 6.6 captive generations (Appendix A). We analyzed the statistical power of our sample size (within captive generations) using leave-one-out rarefaction. During 1,000 iterations of the model, stabilization was achieved in both the slope and R^2^ at a sample of size of 10, suggesting that our sample should be robust enough to detect any possible signal within our data. To evaluate changes in relative endocranial volume across captive generations, we performed a simple linear regression. We calculated the relative difference between the wild population and each individual captive generation using an ANOVA and post-hoc Tukey’s HSD. Captive generations were rounded to whole numbers (including generations 2, 3, 4, 5, and 6) for all ANOVA analyses, therefore the ANOVA analyses do not include captive generation 1. Whereas captive generations remained continuous (from captive generation 1.5 to captive generation 6.6) and were not rounded to whole numbers for linear regression analyses (Appendix A).

Given that the endocranium and the musculature of the skull are not independent of each other, we calculated the bite force of each specimen using Thomason’s dry method (Figure S2), which estimates skull musculature relative to body mass^35,66^. Measurements of the cross-sectional areas, in-lever, and out-lever moment arms of the masseter and temporalis muscles were taken on photos of the dorsal, ventral, and lateral views of the cranium and lateral view of the mandible via Fiji^67^. Specimens that lacked any of these views (n = 2) were not included in the bite force analyses. We assessed the difference between the cross-sectional areas of the masseter and temporalis muscles as well as the overall bite forces of the wild, captive, and reintroduced specimens using an ANOVA and Tukey’s HSD. We also investigated the relative difference in the cross-sectional areas of the masseter and temporalis muscles and in the overall bite force across captive generations using a simple linear regression. Unless otherwise noted, we conducted all analyses in R version 3.6.3^68^.

## Results

The average absolute endocranial volume (+/− 1 standard deviation) of captive, wild, and reintroduced Mexican wolves was 134.12 mL (+/− 9.83 mL), 132.01 mL (+/− 9.39 mL), and 130.31 mL (± 8.49 mL), respectively (Table 1). Captive individuals displayed the greatest overall endocranial volume, which was 1.59% greater than the wild wolves and 2.83% greater than the reintroduced wolves. We did not detect a clear statistical difference in skull length (F = 1.46; df = 2; p = 0.24; Table 1) or endocranial volume (F = 0.95; df = 2; p = 0.39) among captive, wild, and reintroduced groups. Thus, we used a common allometric relationship associated with skull length and endocranial volume (r^2^ = 0.15, F = 15.62, p = 1.6e^−4^; Figure S3) and conducted our analyses using the residual values from this regression. All results were similar regardless of whether the endocranial volumes were calculated relative to skull length or to skull centroid size (which also estimates size by skull length and width measures).

**Table 1:** Mean measures of skull length, endocranial volume, and the residual of the regression of skull length and endocranial volume (+ /- standard deviation) as well as the ANOVA results associated with each.

	Mean measures			ANOVA results		
	Captive (n = 51)	Wild (n = 22)	Reintroduced (n = 12)	F-statistic	df	p-value
Skull length (mm)	20.75 + /− 1.20	20.47 + /− 1.07	20.16 + /− 1.03	1.46	2	0.24
Volume (mL)	134.12 + /− 9.83	132.01 + /− 9.39	130.31 + /− 8.49	0.95	2	0.39
Skull length-volume residual	5.6e−3 + /− 0.07	−5.7e−3 + /− 0.07	−0.01 + /− 0.07	0.46	2	0.63

*P*-value significance: 0.01–0.05*, 0.001–0.01**, 0–0.001***.

**Figure 1:**
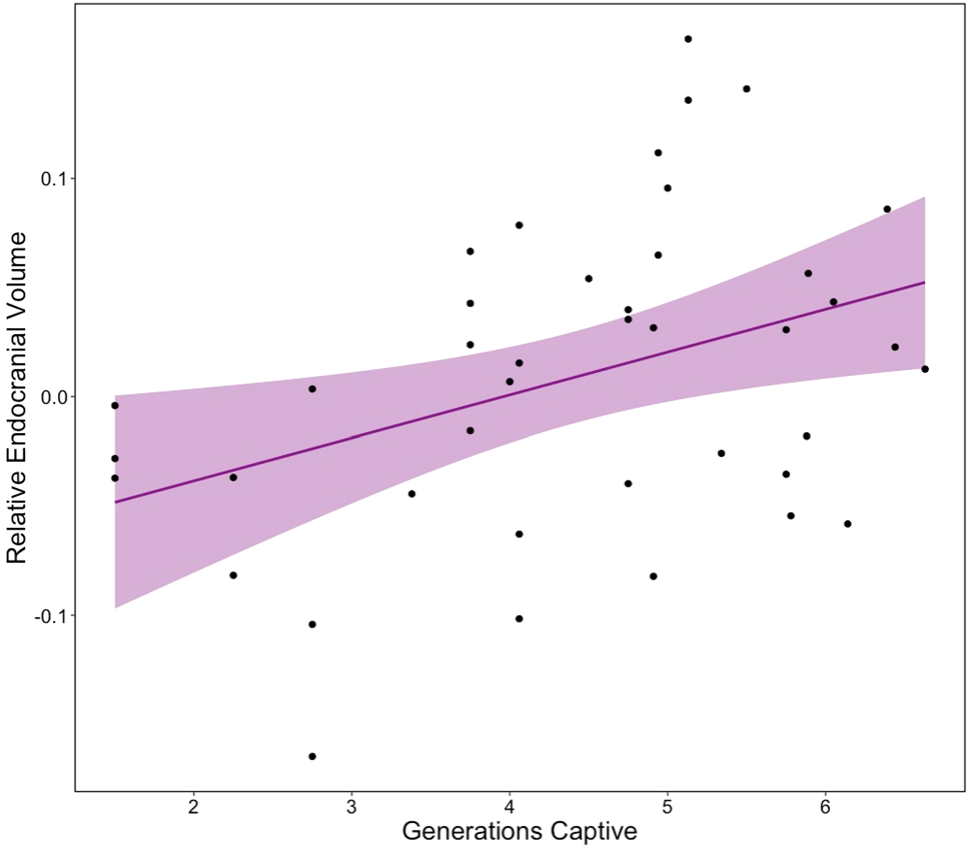
Linear regression of relative endocranial volume (allometrically corrected residuals of endocranial volume relative to skull length) across captive generations, showing a clear trend of increasing volume with subsequent generations (r^2^ = 0.13, F = 6.90, p = 0.01*). Points denote individual specimens; the shaded region represents a 95% confidence interval. *P*-value significance: 0.01–0.05*, 0.001–0.01**, 0–0.001***.

We observed a statistical difference in endocranial volumes across captive generations (r^2^ = 0.13, F = 6.9, p = 0.01*), where generations further removed from the wild population displayed higher mean values of endocranial volume relative to skull length (Fig. 1). This increase peaked at generation 5 (Fig. 1), which was 8.40% greater than the first captive generation recorded in our dataset (captive generation 2, where captive generations were rounded to whole numbers) and was 4.29% greater than the overall mean value for the captive population. Captive generation 2 displayed a smaller endocranial volume than any other captive generation included in this analysis and was 3.94% smaller than the overall mean value for the captive population and 2.30% smaller than the mean value for the wild population. The average length of the skull also decreased across captive generations, where the skull length of the first captive generation (21.17 mm ± 1.11 mm) was 4.40% longer than average length of captive generation 6 (20.23 mm ± 1.13), however; this trend did not result is a statistical difference (r^2^ = 0.04, F = 2.5, p = 0.12).

**Figure 2:**
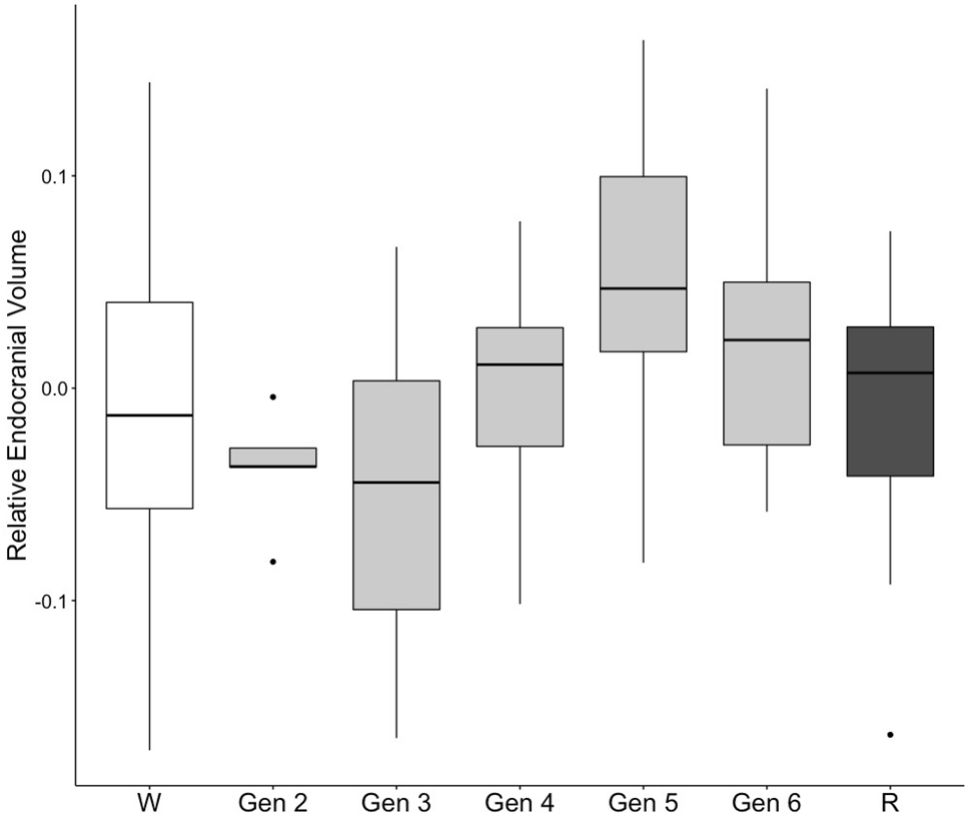
Relative endocranial volume (allometrically corrected residuals of endocranial volume relative to skull length) for wild (W), reintroduced (R), and captive generations rounded to whole numbers including captive generation 2 (Gen 2) 3 (Gen 3), 4 (Gen 4), 5 (Gen 5), and 6 (Gen 6). No clear statistical differences were observed between groups (F = 0.46; df = 2; p = 0.63), however captive wolves had greater variability. White represents wild, gray represents captive, and black represents reintroduced specimens. *P*-value significance: 0.01–0.05*, 0.001–0.01**, 0–0.001***.

We detected a statistical difference between the captive generations and the reintroduced population (F = 2.48; DF = 5; p = 0.04*; Fig. 2). In particular, the endocranial volume of captive generation 5 was 6.84% greater than the reintroduced population; however, the Tukey HSD analysis did not detect a pairwise difference between any of the captive generations and the reintroduced group, including comparisons with the largest overall captive generation (captive generation 5; t = − 2.32; p = 0.20). We also detected a significant difference between the wild group and the captive generations (F = 2.95; DF = 5; p = 0.02*), where captive generation 5 was 5.96% greater than the overall wild population average. However, Tukey HSD analysis did not detect any pairwise differences between the captive generations and the wild group (Fig. 2).

We did not detect a difference in estimated bite force either across captive generations (r^2^ = 0.05, F = 3.75, p = 5.8^e−2^) or between the captive, wild, and reintroduced populations (F = 2.03, Df = 2, p = 0.14). Although the reintroduced population showed the weakest overall bite force, which was 8.34% weaker than the captive population and 11.18% weaker than the historic wild population. We detected a difference in the cross-sectional area of the masseter relative to the jaw length across captive generations (r^2^ = 0.08, F = 5.66, p = 0.02*), associated with a decrease over captive generations (Figure S4). We also detected a difference in the masseter cross-sectional area between the captive, wild, and reintroduced populations (F = 4.76, Df = 2, p = 0.01*). Tukey’s HSD detected a pairwise difference between the masseter cross-sectional area of captive and reintroduced specimens (t = − 2.82, 0.02*), where captive individuals displayed the greatest cross-sectional area overall (Figure S5). We did not detect a difference in the cross-sectional area of the temporalis muscle either across captive generations (r^2^ = − 2.8e^−3^, F = 0.86, p = 0.36) or between captive, wild, and reintroduced populations (F = 2.39, Df = 2, p = 0.10).

We did not detect a relationship between endocranial volume and bite force (r^2^ = − 0.01, F = 0.18, p = 0.67) nor did we detect a relationship between endocranial volume and the cross-sectional areas of either the masseter or the temporalis muscles (r^2^ = 3.9^e−3^, F = 1.32, p = 0.25 and r^2^ = − 3.6^e−3^, F = 0.71, p = 0.40, respectively). Similarly, we did not detect a relationship between the relative endocranial volume and the inbreeding coefficients of the captive population (r^2^ = − 0.01, F = 0.71, p = 0.41).

## Discussion

In direct opposition to the expected trend, we show an increase in the endocranial volume of Mexican wolves across captive generations. Given the results of previous studies regarding captive-bred animals, which have typically either reported a decrease in endocranial volume in captivity [e.g., ^7,12,23^] or no discernable difference in captive populations^10,69^, we expected to find similar results for captive Mexican wolves. This trend toward an increasing endocranial volume may suggest that the relationship between captivity and brain size is more complicated and less consistent than previously thought and will require a more nuanced approach in future analyses.

Species that have undergone significant population bottlenecks may be particularly prone to a reduction in endocranial volume^12,31^. Given that the captive population of Mexican wolves was founded with just seven animals^56,57^, we anticipated a correlation between relative endocranial volume and inbreeding; however, we detected no such relationship. It is important to note that inbreeding coefficients calculated exclusively from studbook data may underestimate the full extent of trends as they cannot account for inbreeding that occurred prior to captivity^70^. However, the inbreeding coefficients calculated here have been previously validated against inbreeding estimates calculated from genetic data^62,71^. Regardless, an association with inbreeding seems unlikely in this case given the increase in endocranial volume across captive generations. Notably, however, the first captive generation in our dataset displayed the smallest overall endocranial volume, which was not only smaller than any other captive generation, it was also smaller than both the wild and reintroduced population means. This may reinforce the idea that the founding captive population experienced poor overall health due to the bottleneck they experience prior to forming the captive population.

We did not detect a clear difference in bite forces either among captive, wild, and reintroduced Mexican wolves or across captive generations, although we did detect a difference associated with the cross-sectional area of the masseter muscle, which suggests that the masseter may be stronger within the captive population. This change may be unsurprising given that the primary attachment site for the masseter muscle occurs from the mandibular masseteric fossa to the zygomatic arches^72^ and captive Mexican wolves display comparatively wide skulls^62^, providing additional space for muscle attachment. However, if the trend toward increasing endocranial volume across captive generations was related to the constraints of cranial musculature upon cranial shape, as hypothesized here, we might expect a weaker temporalis muscle associated with the captive population given that the primary attachment site of the temporalis occurs on the braincase^72^. The fact that we did not detect a difference in the temporalis muscle either among captive, wild, and reintroduced groups or across captive generations suggests that either the temporalis muscle has not changed in conjunction with cranial morphology or that the measurement technique used here may be too coarse to detect the changes. We encourage future studies to explore this relationship more closely using soft tissue analyses.

The literature exploring shifts in endocranial volume of captive animals has mostly focused on fish [e.g.,^8,12,22^] or non-carnivorous mammals [e.g.,^10,69^]. These studies use animals reared in captive situations where animal husbandry (e.g., enrichment) may be a lower priority, such as lab-reared species [e.g.,^24,25^], or those with a captive history that extends to a time prior to the establishment of strict husbandry protocols [e.g.,^7,30^]. However, captive husbandry has improved significantly over the past century with the oversight of accrediting organizations, which monitor aspects of animal welfare including nutrition, enrichment, and enclosure design^73^. The more recent captive history represented by the Mexican wolves in this study (which only date back to 1975) may suggest that modern efforts to improve animal welfare may have led to an increase in brain size. Even by modern standards, captive Mexican wolves may receive above average care given their status as a remnant population of an endangered species bred specifically for conservation purposes.

Captive management may have a strong effect on endocranial volume. The development of brain tissue, for example, is facilitated by high-nutrient diets, where malnourished animals may experience poor brain development^47,74^. While high-quality diets are generally provided on a daily basis in captivity, the availability of consistently high-quality diets may be difficult to come by in the wild. The differential nutrition available in captivity may therefore suggest that captive animals should be expected to display an increase in endocranial volume compared to their wild counterparts, particularly among more recently established captive populations. For example, Hecht and colleagues (2021)^75^ found that regardless of whether they were bred for aggression or docility, silver foxes (*Vulpes vulpes*) maintained in a provisioned captive environment developed larger brain sizes than their farm-bred counterparts.

Other aspects of captive management, including enclosure design and the availability of enrichment, have been linked to changes in brain size^22,40^. For example, rats maintained in enriched enclosures display significantly larger brain sizes than those without enrichment^76,77^. Similar findings have also been associated with a lack of appropriate social stimulation^8,78^. Given that they were bred to initiate a reintroduction program, captive Mexican wolves may have avoided some of these drawbacks as special care was likely taken to breed healthy animals that maintained species-appropriate behaviors. Future studies should explore the nature of these trends in terms of captive husbandry and management, particularly nutrition, enclosure design, and enrichment.

Although we detected a significant increase in endocranial volume across captive generations, we did not detect a significant difference between captive, wild, and reintroduced groups. This may be related to the variation in endocranial volume associated with the captive population. Although captive generation five displayed the largest endocranial volume overall, the first captive generation in our dataset displayed a lower mean endocranial volume than either the wild or reintroduced groups, potentially obscuring some of the trends in the data. The endocranial volume of captive generation five was 6.84% larger than the reintroduced population and 5.96% larger than the wild population, suggesting that the peak endocranial volume achieved in captivity may have been quickly lost after the animals were reintroduced to their wild habitat.

Our results suggest an unexpected trend in the endocranial volume of captive Mexican wolves in which brain size increases over successive captive generations. Given that this increase appears to be absent from reintroduced populations of these wolves, these trends are likely to be related to the enhanced provisions accessible in captivity including consistently high-quality nutrition. These results suggest that a loss of brain volume in captivity is no longer a clear expectation and that with proper captive management and careful husbandry decisions, brain volumes may be maintained or actually increase. Intensive captive management of endangered species is common and may be fraught with a variety of unintended negative consequences; however, the results of this study suggest that a loss of brain volume need not be a foregone conclusion.

## Supplementary Information

Supplementary Information 1.

Supplementary Information 2.

## Data Availability

All data analyzed during this study are included in this published article (and its appendix file).
