# Supplementary material for: Endocranial volume increases across captive generations in the endangered Mexican wolf
Source: Sci Rep. 2022 May 17;12:8147. doi: 10.1038/s41598-022-12371-6 (PMC9114419; doi:10.1038/s41598-022-12371-6)

Supplementary Tables & Figures

Figure S1: Landmark scheme used to calculate the skull centroid size.


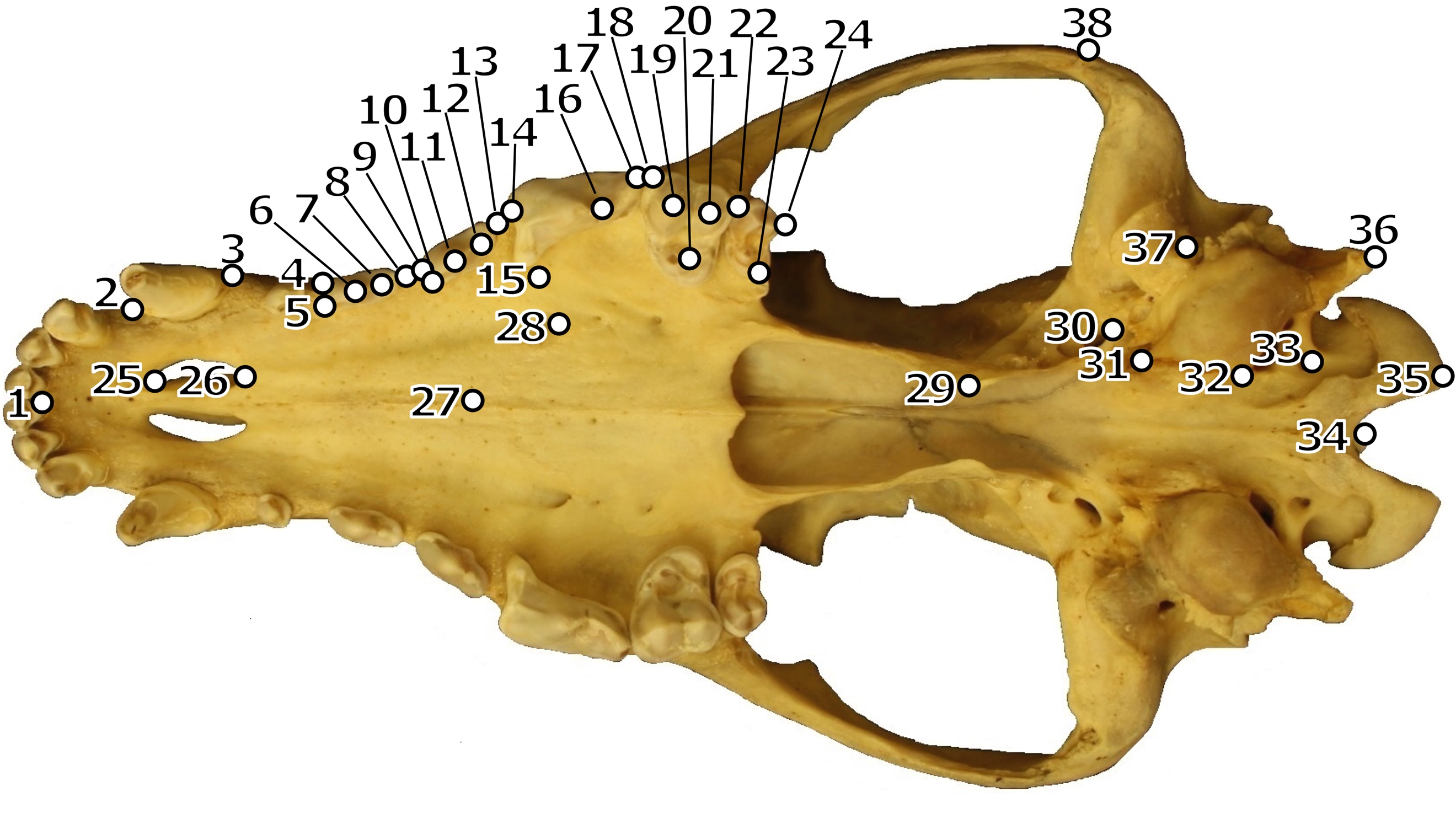


Figure S2: The cross-sectional areas of the masseter (M) and temporalis (T) muscles were measured. For each muscle, the distance from the centroid of the muscle cross section to the joint was measured as the input lever arm distance (dM and Td respectively). The output lever arm was measured from the condyloid process of the mandible to the shearing facet of the lower carnassial (dO). Total skull length (L) and Bite force (F) was calculated according to Thomason’s dry-skull procedure (Thomason 1991) as:

$$F=\frac{(d_{M}\times\left\{ M\times300 \right\}+d_{T}\times\left\{ T\times300 \right\})}{d_{o}}$$

To correct for estimates done on the dry skulls and standardize by body mass calculated from skull length (L), the following correction equation (Damasceno et al., 2013) was applied:

$$BFQ=\frac{F_{corr}}{F_{L}}=\frac{{10}^{(0.859\times\log F+0.559)}}{{10}^{(1.95\times logL-1.12)}}$$

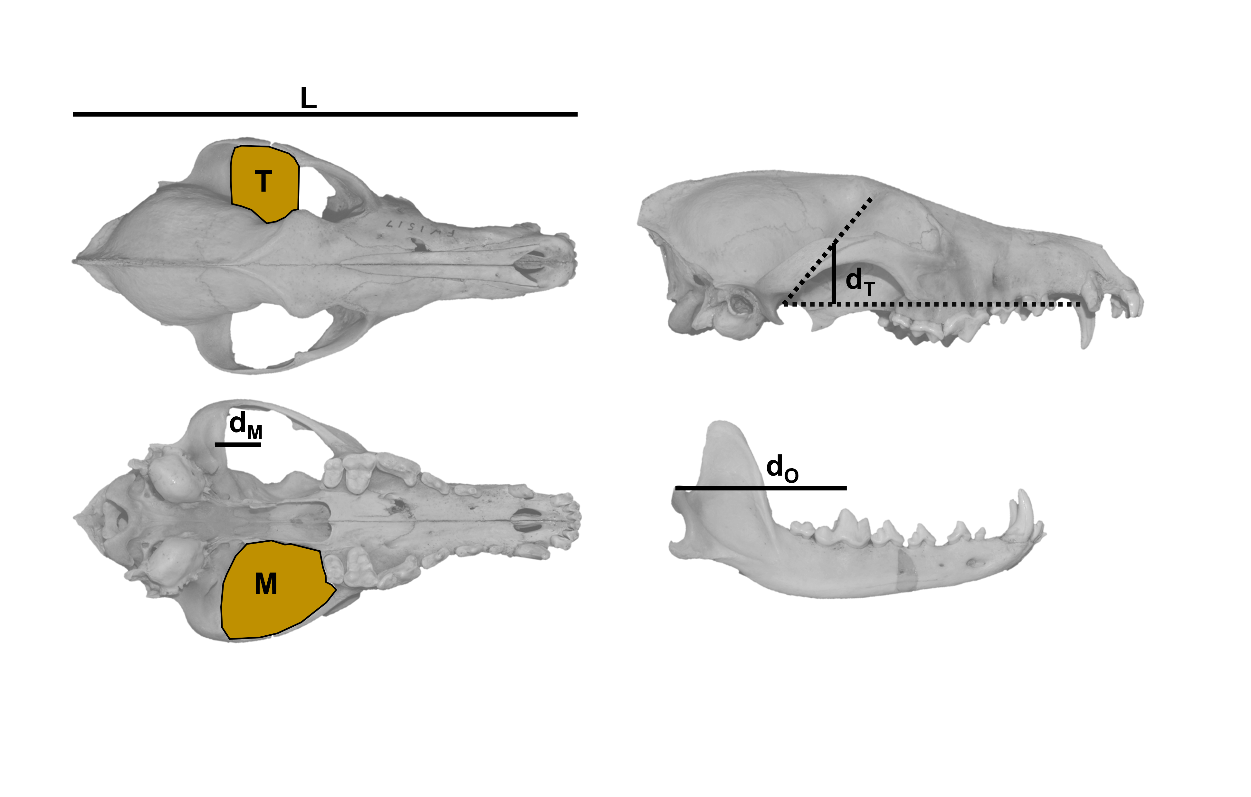


Figure S3: A. Linear regressions of endocranial volume (mL) relative to skull length (mm) and B. the skull centroid size. Red represents captive, green represents reintroduced, and blue represents wild.


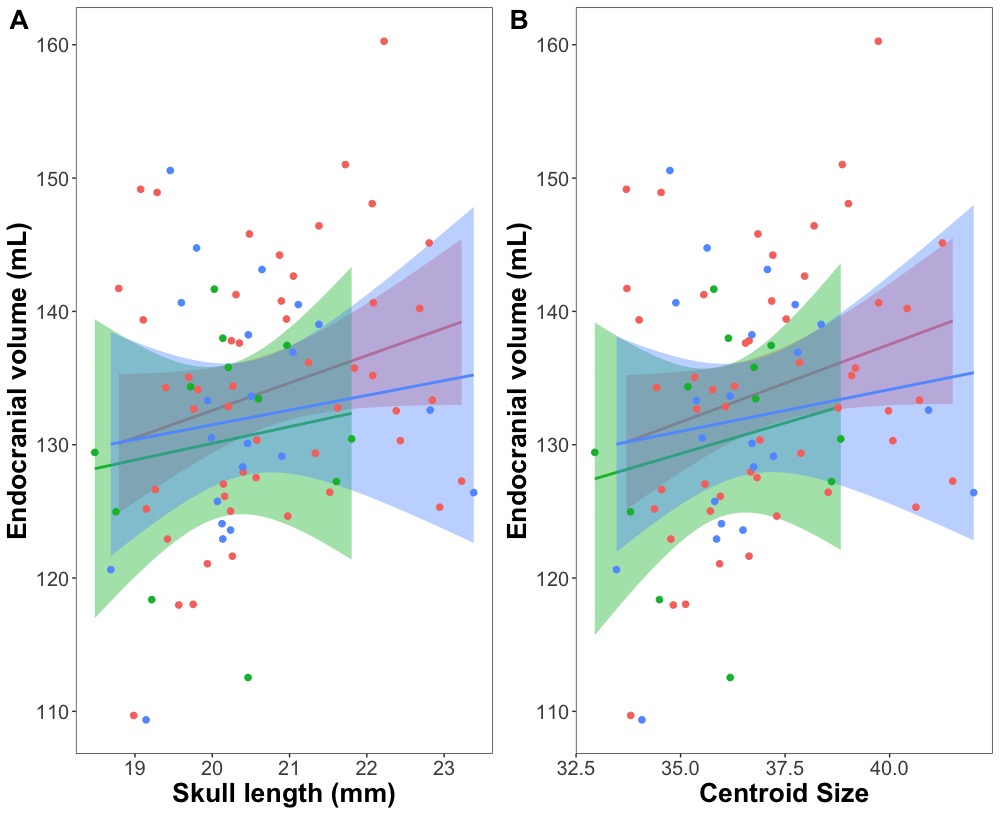


Figure S4: Masseter muscle cross-sectional area (mm^2^) relative to jaw length (mm) in across captive generations of Mexican wolves.


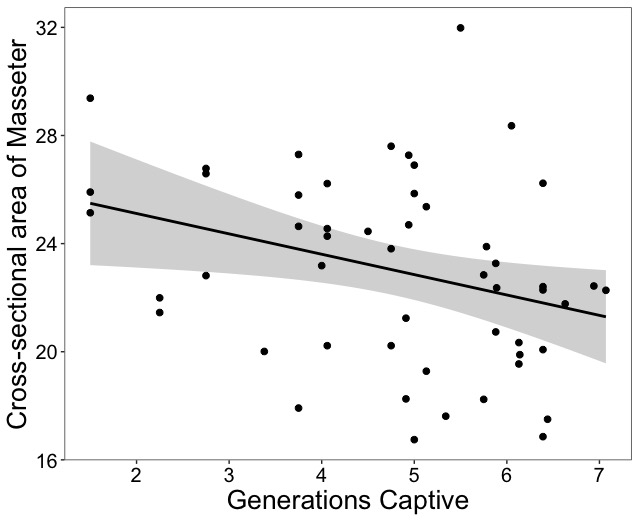


Figure S5: Mean masseter muscle cross-sectional area (mm^2^) relative to jaw length (mm) in captive (red), wild (green), and reintroduced (blue) Mexican wolves.


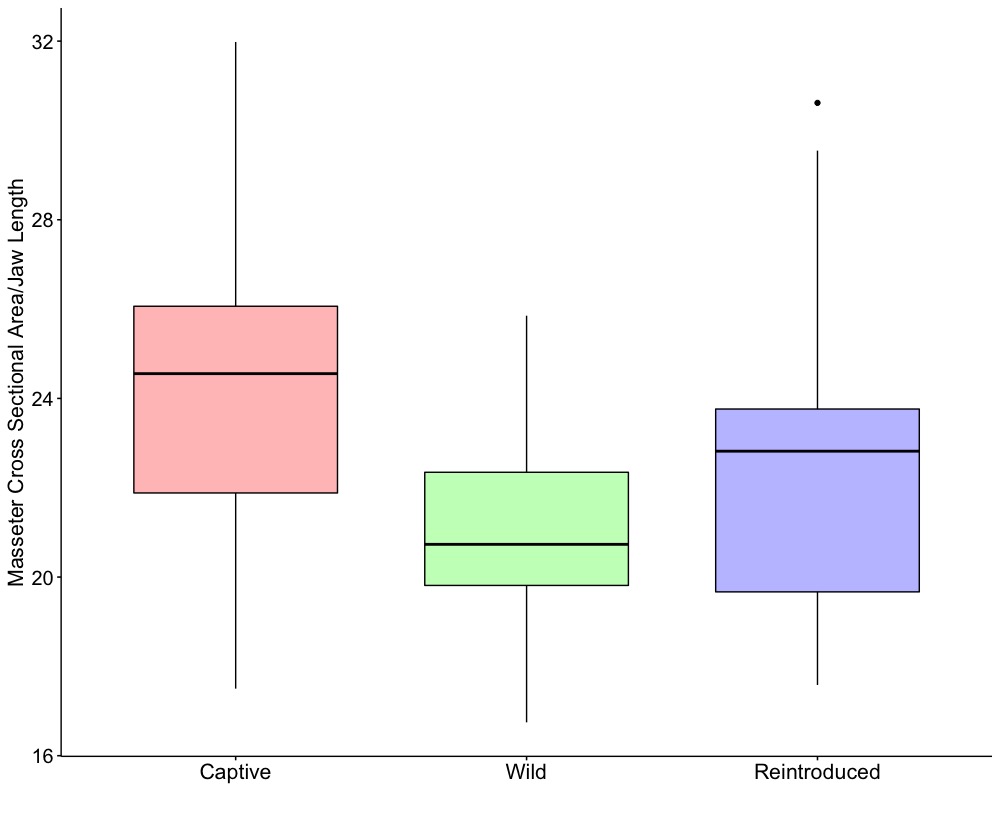

Supplement: Supplementary file 2 — Supplementary Information 2. [file 41598_2022_12371_MOESM2_ESM.docx]
